# The Assessment of Myocardial Strain by Cardiac Imaging in Healthy Infants with Acute Bronchiolitis: A Systematic Review and Meta-Analysis

**DOI:** 10.3390/diagnostics10060382

**Published:** 2020-06-08

**Authors:** Moises Rodriguez-Gonzalez, Alvaro Antonio Perez-Reviriego, Ana Castellano-Martinez, Helena Maria Cascales-Poyatos

**Affiliations:** 1 Pediatric Cardiology Division, Puerta del Mar University Hospital, 11009 Cadiz, Spain; doctormoisesrodriguez@gmail.com (M.R.-G.); alvaro.apr@hotmail.com (A.A.P.-R.); helena.mcp@hotmail.com (H.M.C.-P.); 2 Biomedical Research and Innovation Institute of Cadiz (INiBICA), Research Unit, Puerta del Mar University Hospital, University of Cadiz, 11009 Cadiz, Spain; 3 Pediatric Nephrology Division, Puerta del Mar University Hospital, 11009 Cadiz, Spain

**Keywords:** acute bronchiolitis, respiratory syncytial virus, echocardiography, tissue doppler imaging, speckle-tracking echocardiography, myocardial strain, pulmonary hypertension, NT-proBNP, troponin, children

## Abstract

This study aims to systematically review the incidence of myocardial strain detected by echocardiography in previously healthy infants with acute bronchiolitis and its role as a predictor for adverse outcomes in this setting. Methods: Pubmed/Medline, Excerpta Medica Data Base (EMBASE), and Cochrane Library were searched in April 2020 to identify original observational prospective studies that systematically performed echocardiography for the screening of myocardial strain in healthy infants with acute bronchiolitis. Pooled estimates were generated using random-effects models. Heterogeneity within studies was assessed using Cochran’s Q and I^2^ statistics. Funnel plots and Egger´s regression method were constructed to evaluate publication bias. Sensitivity analyses were also conducted to evaluate potential sources of heterogeneity. Results: After a detailed screening of 305 articles, a total of 10 studies with 395 participants (mean of 40 participants per study) was included. Five of them were classified as high-quality studies. Up to 28% of cases presented adverse outcomes. The echocardiographic screening for myocardial strain was performed within the first 24 h of admission in 92% cases. Tissue Doppler imaging and Speckle-Tracking echocardiography were performed only in 20% of cases. The presence of pulmonary hypertension was evaluated with methods different from the tricuspid regurgitation jet in 64% of cases. Seven studies found some grade of myocardial strain with a pooled incidence of 21% (CI 95%, 11–31%), in the form of pulmonary hypertension (pooled incidence of 20% (CI 95%, 11–30%)), and myocardial dysfunction (pooled incidence of 5% (CI 95%, 1–9%)). The presence of these echocardiographic alterations was associated with adverse outcomes (pooled relative risk = 16; CI 95%, 8.2–31.5). After a subgroup analysis based on the echocardiographic techniques used, no significant heterogeneity across the studies was observed. There was no evidence of publication bias when assessed by Egger´s test. Cardiac biomarkers to assess myocardial strain were used in five studies. Only N-terminal-pro-brain natriuretic peptide accurately predicted the presence of myocardial strain by echocardiography. Conclusions: Myocardial strain is not infrequent in previously healthy infants with acute bronchiolitis, and it could be present at the early stages of the disease with prognostic implications. There is a need for sufficiently powered prospective studies with a similar methodology, preferably employing advanced imaging techniques, to conclusively address the usefulness of the assessment of myocardial strain in this setting.

## 1. Introduction

Acute bronchiolitis (AB) is the leading cause of lower respiratory infection and hospitalization among children by the age of 2 years worldwide [1,2,3,4]. Severe AB constitutes a significant pediatric intensive care unit (PICU) burden. Approximately 5% of cases of AB require PICU admission, and any type of advanced respiratory support (ARS) [2,4,5]. Therefore, it is crucial to identify those patients at highest risk of severe disease in order to provide the best management options and potentially decrease morbidity. Current guidelines recommend the identification of specific risk factors and clinical assessment as the best tools to assess severity, predict evolution, and tailor management accordingly [6]. There are certain high-risk groups (premature infants, bronchopulmonary dysplasia, congenital heart disease (CHD), immunocompromised, Down syndrome) that are associated with an increased vulnerability to AB [6,7,8]. However, numerous studies have also demonstrated that a majority of children hospitalized with AB lack these risk factors and were healthy before their clinical event; of note, nearly half of children admitted to PICU with severe AB were previously healthy [1,2,4,7,9,10]. Furthermore, the clinical scores employed to assess the clinical severity in AB have a considerable inter-observer and intra-observer variability that limits their application. They have no predictive value, and they only assess the clinical state at the moment of the evaluation [9,11,12,13]. As a result, efforts aimed at developing an effective method to predict outcomes at the early stages of the disease have multiplied.

Cardiovascular involvement in patients with AB is well known and especially concerning. In patients with CHD, the increased risk of severe disease may be related to multiple physiological factors including baseline compromised cardiorespiratory function, changed mechanisms of pulmonary regulation, ventilation-perfusion mismatch, and potential cyanosis, pulmonary hypertension (PH), and myocardial dysfunction (MD) [7,14,15]. Interestingly, cardiovascular involvement has also been observed in previously healthy infants with AB. Thus, cardiovascular complications (arrhythmias, extreme bradycardia, cardiac tamponade, sepsis-like episodes, MD, shock with the need for inotropic support) are reported in up to 9% of cases of AB and constitute the second most common extrapulmonary manifestations in AB [16]. Of note, these cardiovascular manifestations are usually described in those children with severe AB requiring PICU admission or mechanical ventilation [16,17,18,19,20].

Infants with respiratory insufficiency from bronchiolitis may have similar clinical features to infants with heart failure, presenting with irritability, fever, tachypnea, tachycardia, and a mottled appearance. Therefore, the cardiovascular evaluation of infants with AB based only on the physical examination is challenging, and it would be better assessed by other methods. To date, no data regarding myocardial strain (MS) assessed by cardiac imaging in infants with AB have yet been systematically collected. This systematic review and meta-analysis aim to investigate and summarize the current evidence for the incidence and types of MS in previously healthy infants with AB. Furthermore, this study aims to investigate the role of MS as a predictor for adverse outcomes in this population.

## 2. Materials and Methods 

This systematic review follows the Preferred Reporting Items for Systematic Review and Meta-Analyses (PRISMA) guidelines and is reported following the PRISMA statement.

### 2.1. Search Strategy

A systematic search was conducted independently by two authors (M.R.-G. and A.A.P.-R.) on PubMed/MEDLINE, COCHRANE, and EMBASE databases for all publications with a focus on the relationship between MS and AB in children reported from January 1980 to April 2020. The following search terms were used: (acute bronchiolitis OR respiratory syncytial virus) AND (echocardiography OR pulmonary hypertension OR ventricular dysfunction).

### 2.2. Selection Criteria

After clearance of duplications of the retrieved citations, the titles and abstracts of the remaining English articles were screened for relevance based on the established selection criteria. Two authors (M.R.-G. and A.A.P.-R.) considered inclusion and exclusion independently. Inconsistency was solved by discussion. In all initially selected articles, references were crosschecked using the same inclusion and exclusion criteria to identify relevant articles missed by the initial literature search for potential inclusion. 

#### 2.2.1. Inclusion Criteria

- AB diagnosed following the recommendations of international guidelines [6].
- Age younger than 2-year-old. Children without CHD.
- Systematic echocardiography for screening of MD and PH.

#### 2.2.2. Exclusion Criteria

- Adult or animal studies.
- Investigations of other diseases than AB.
- Presence of well-known risk factors for severe AB, such as CHD.
- Studies in which echocardiography was performed for clinical indications.
- Pilot studies, retrospective studies, multiple papers from the same study and not primary research (reviews, letters, guidelines, case reports, case series).

### 2.3. Data Extraction 

One author (A.C.-M.) extracted the following information from each article: name of the first author, year of publication, design of the study, sample size (specifying the number of cases with MS and controls), the incidence of severe clinical AB (high clinical score, hypoxemia or respiratory acidosis) or adverse outcomes (PICU admission or advanced respiratory support), the echocardiographic technique used to assess MS, the timing and setting of echocardiography, type of cardiac biomarkers and their values in assessing MS, the incidence and type of echocardiographic alterations found (MD and PH), associations between echocardiographic alterations and adverse outcomes, associations between cardiac biomarkers and echocardiographic alterations, statistical details and relevant results of the study. When stated in the article, the detection rate of MD and PH by echocardiography and the relative risk (RR) and 95% confidence interval (CI 95%) for adverse outcomes or severe AB were extracted from the papers. If these data were not in the articles, the detection rates and RR (CI 95%) were calculated on a per-patient-based analysis by the same author (A.C.-M.) All data were independently checked by a second author (H.M.C.-P.); disagreements were resolved by discussion. For studies without enough quantitative data, the correspondent author was contacted, and if no answer was obtained, the studies were excluded. 

### 2.4. Quality Assessment

Two unblinded reviewers (M.R.-G. and A.A.P.-R.) independently evaluated the methodological quality of the included observational studies using a modified version of the Downs and Black evaluation tool. The tool consists of 27 questions across five sections: study quality (ten items), external validity (three items), internal validity bias (seven items), confounding selection bias (six items), and power of the study (one item) with an overall score out of a possible 32 points. In the present version of the checklist we modified the scoring of item 27 that refers to the power of the study. Instead of rating according to an available range of study powers, we rated whether the study performed power calculation or not. Accordingly, the maximum score for item 27 was 1 (a power analysis was conducted) instead of 5 and thus the highest possible score for the checklist was 28 (instead of 32). Downs and Black score ranges were given corresponding quality levels as excellent (26–28); good (20–25); fair (15–19); and poor (<14). Discussion resolved the disagreements between the reviewers, with the involvement of a third review author where necessary (H.M.C-P.). 

### 2.5. Statistical Analysis

Continuous variables were reported as mean (standard deviation) or median (interquartile rank). Categorical variables were expressed as number and percentage (%). Multiple separate meta-analyses were conducted to calculate the combined detection rate of MD and PH, and RR for severe AB. Considering the high likelihood of between-study variance, a random-effects model was used. Continuity correction for total zero events studies was performed to include these in the meta-analysis as recommended. Pooled data were presented with 95% confidence intervals (95% CI) and displayed using forest plots. Heterogeneity within studies was assessed using Cochran’s Q and I^2^ statistics. If the *P*-value for heterogeneity was determined to be <0.10 or the I^2^ value was >50%, the presence of heterogeneity was taken into consideration, and exploratory subgroup analyses were performed to identify potential causes of heterogeneity. Funnel plots and Egger’s regression method were constructed to evaluate publication bias. Sensitivity analysis was performed to assess the influence of each individual study on the estimated effects by omitting individual studies. All of the tests were two-tailed with alpha being set at 0.05, except for the homogeneity test, whose significance level cut-off was considered to be 0.10 due to the low power of the χ2 test with a limited amount of studies. All the statistical analyses were performed using the STATA 14.0 (StataCorp. College Station, TX, USA). 

## 3. Results

### 3.1. Eligible Studies

**Figure 1:**
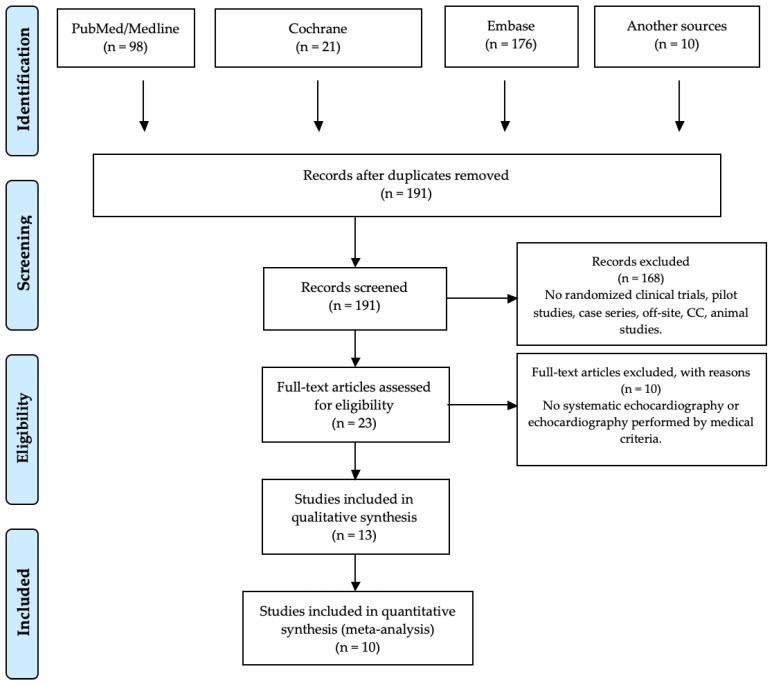
PRISMA 2009 flow diagram showing the methodology of the search performed for this systematic review.

The literature search yielded 305 citations. After clearance of duplications, 191 articles remained potentially useful. Based on the title and abstract, 168 articles were excluded. Ten articles appeared not to have a full text available. Evaluation of the full texts of the remaining 23 articles led to the exclusion of 11 articles. Ultimately, 10 articles were included in this systematic literature review and meta-analysis [21,22,23,24,25,26,27,28,29,30] (Figure 1). Two studies were carried out by the same authors [28,29], but with two different cohorts, and therefore there were no overlapping data. 

### 3.2. Quality Assessment of the Included Studies

All 10 selected studies consisted of observational prospective cohort studies; three of them had control subjects [25,29,30]. The ratings of the quality of the methods of the individual studies are presented in Table 1. Overall, the scores were generally fair, with only three out of the ten studies achieving a good methodological quality [28,29,30]. Studies typically lost points for internal validity and confounding and selection bias because of questions which were aimed at randomized and intervention trials. Four studies [23,24,26,27] did not provide a representative of the study population; two of them [23,26] included participants with severe AB at the moment of the enrollment, and one of them [27] utilized a convenience sample. Only five studies [24,27,28,29,30] could demonstrate that severe disease or adverse outcomes were not present at the start of the study. Comparability of the cohorts was possible in seven studies [21,24,25,27,28,29,30]. Two studies could not provide adequate information about outcomes [23,26].

### 3.3. Qualitative Analysis

#### 3.3.1. Baseline Characteristics

The details of the study characteristics are summarized in Table 2. There were 395 infants enrolled in 10 studies. The sample size of the investigations was small in most cases (mean size of 40 (26) patients per study). Only four studies [24,25,28,29] included more than 50 participants. Almost all participants were previously healthy infants younger than two-years-old. The mean/median age of most studies was less than five-months-old. Two studies [21,22] did not detail the exact mean/median age of their participants, only the range (1–24 months) We only detected two cases of cystic fibrosis, four cases of premature newborns and one case of Down syndrome as not previously healthy infants in three studies [21,22,23]. We decided not to exclude these articles because these cases constituted only a minimal part of the pooled cohort (1%). Remarkably, there were no infants with CHD included in any of the selected studies, as this was one of the exclusion criteria. 

#### 3.3.2. Assessment of Myocardial Strain

##### Echocardiographic Timing and Techniques

The echocardiographic screening for MS was performed within the first 24 h of admission in seven studies [22,24,25,27,28,29,30] that included 92% of all the cases included in the systematic review (Table 2). In one study [26], 80% of echocardiograms were performed within 24 h of admission, and the rest were performed within 48 h. In the studies that took the longest to perform an echocardiogram [21,23], it was done after 72 h of admission. We identified different echocardiographic techniques that had been employed for the detection of MD and PH from the included studies (Table 3). All authors used conventional echocardiography to assess MD. Advanced imaging techniques to assess MD, such as Tissue Doppler imaging (TDI) and Speckle-Tracking echocardiography (STE), were performed only in two investigations [29,30], accounting for 20% of cases included in the systematic review. The parameters used to assess the left ventricular function were left ventricular fractional shortening (LVFS), left ventricular ejection fraction (LVEF), mitral annular plane systolic excursion (MAPSE), the myocardial performance index (MPI) or Tei index and the longitudinal (LS), circumferential (CS) and radial strain (RS). The parameters used to assess the right ventricular function were the tricuspid annular plane systolic excursion (TAPSE), the fractional area change (FAC), and the MPI of the right ventricle (RV). One study evaluated the left and right ventricular cardiac output by 2DDoppler Echo (29). The parameters used to assess PH were the tricuspid regurgitation jet gradient (TRJG), systolic duration to diastolic duration ratio, pulmonary flow analysis, septal flattening (SF), left ventricular eccentricity index (LVEI), and pulmonary acceleration time/right ventricular ejection time ration (ATET). Six studies [21,22,23,24,26,27] used only the TRJG method to assess PH), while four studies [26,28,29,30] evaluated the presence of PH combining different echocardiographic parameters, accounting for 55% of cases included in the systematic review.

##### Cardiac Biomarkers

The investigators utilized cardiac biomarkers (troponin I, troponin T, or NT-ProBNP) to assess MS combined with echocardiography in five studies [24,26,27,28,29] (Table 4), accounting for 64% of cases included in the systematic review. In one study [28] the authors utilized both troponin T and NT-ProBNP. None of the investigated cardiac biomarkers have been sufficiently studied in the same way, hampering further comparison or meta-analysis. An elevated level of cardiac biomarkers was found in four studies [24,26,27,28]. Increased troponin and NT-proBNP levels were observed in 14–41% and 25% of cases, respectively. Only in one study [28], was the troponin associated with echocardiographic findings (PH), but not in the multivariate analysis. NT-proBNP resulted in an accurate and independent predictor for both PH (AUC = 0.932; cutoff point 1345 pg/mL yielded a sensitivity of 0.86, specificity of 0.89; PPV of 0.70 and NPV of 0.95); *p* < 0.001) [28], and MD ((AUC = 0.91; cutoff point 1500 pg/mL yielded a sensitivity of 0.80, specificity of 0.95, a PPV of 0.80, and an NPV of 0.95) [29].

#### 3.3.3. Research Outcome and Incidence of Severe AB

The research outcome used to classify participants as severe AB was the clinical severity (presence of higher clinical scores, hypoxemia, and respiratory acidosis) in five studies [21,24,25,27,30] (48% of cases) and the need of PICU admission or ARS in the remaining studies (52% of cases) [22,23,26,28,29]. Two articles included only patients with mild/moderate AB that did not require PICU [24,26]. Conversely, another two studies only included patients with severe AB and with ARS or PICU admission at the time of the intervention [23,26]. Based on these criteria, a total of 112/395 (28%) of participants in this systematic review presented severe AB. 

### 3.4. Quantitative Analysis

#### 3.4.1. Incidence and Type of Myocardial Strain in AB

**Figure 2:**
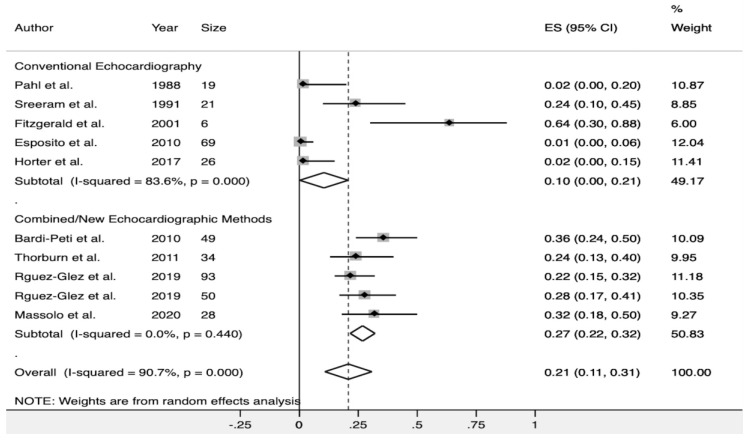
Forest plot showing the subgroup analysis for the detection rate of any type of echocardiographic alteration (myocardial dysfunction or pulmonary hypertension) in healthy infants with AB. Combined methods refers to the use of more than one echocardiographic method (different to tricuspid regurgitation jet gradient) to assess pulmonary hypertension. New Methods refer to the myocardial performance index (Tei index) by conventional or tissue Doppler imaging, or the use of speckle-tracking echocardiography to assess myocardial dysfunction.

The detection of any type of echocardiographic alteration (PH, MD, or both) was reported in seven studies [22,23,25,26,28,29,30] (Table 3), with a pooled estimate measure of 21% (CI95% 11–31%). There was significant heterogeneity among these studies (Q test; I^2^ = 90.7%, *p* < 0.001). A subgroup analysis was performed based on the echocardiographic methods used to assess the MS. After excluding those studies that only used conventional echocardiographic methods to assess myocardial function (LVEF, LVFS or TAPSE), and those that only used 1 echocardiographic method to assess PH (TRJG), the pooled incidence of MS was higher (27% CI 95%, 22–32%), and no significant heterogeneity across the studies was observed (I^2^ = 0.0%; *p* = 0.440) (Figure 2). There was no evidence of publication bias when assessed by Egger’s test (*p* = 0.809).

##### Incidence of Pulmonary Hypertension

**Figure 3:**
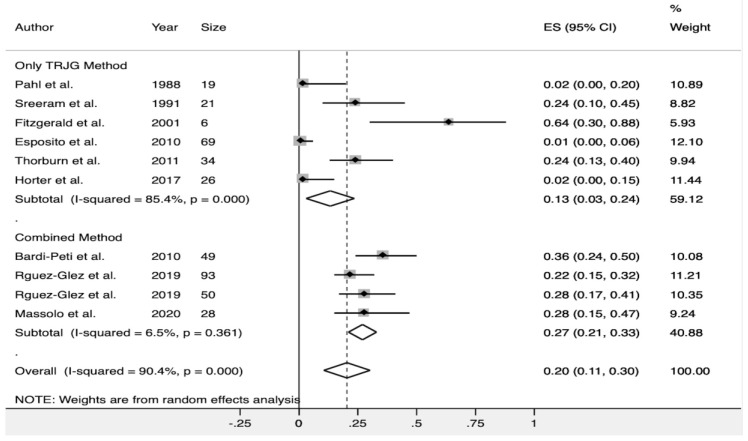
Forest plot showing the pooled detection rate of pulmonary hypertension in healthy infants with AB. Combined methods refers to the use of more than one echocardiographic method (different to tricuspid regurgitation jet gradient) to assess pulmonary hypertension. The subgroup analysis demonstrated that using combined parameters resulted in a higher and consistent detection of pulmonary hypertension in this setting.

Seven authors [22,23,25,26,28,29,30] detected PH with a pooled estimate measure of 20% (CI 95%, 11–30%). There was significant heterogeneity among these studies (Q test; I^2^ = 90.4%, *p* < 0.001). A subgroup analysis was performed based on the echocardiographic methods used to assess PH. After excluding those studies that only used the TRJG method to assess PH, the pooled incidence of PH was higher (27% CI 95%, 21–33%), and no significant heterogeneity across the studies was observed (I^2^ = 6.5%, *p* = 0.361) (Figure 3). There was no evidence of publication bias when assessed by Egger’s test (*p* = 0.921).

##### Myocardial Dysfunction

**Figure 4:**
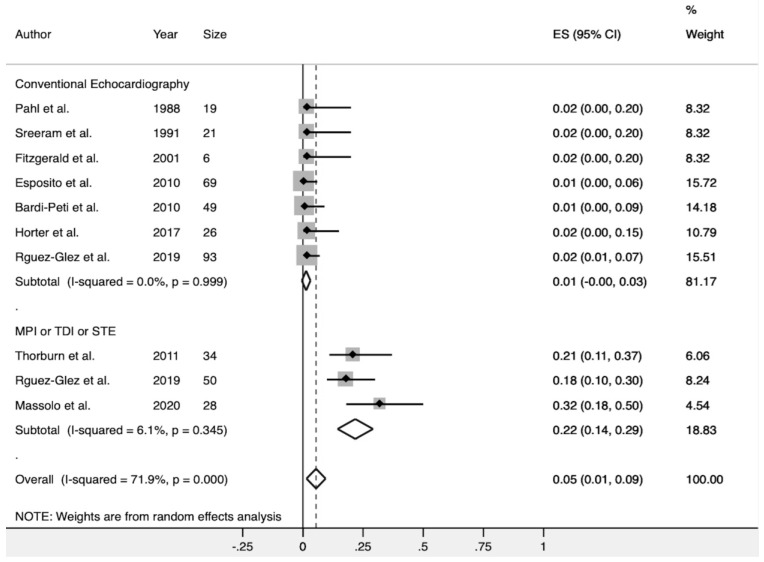
Forest plot showing the combined detection rate of myocardial dysfunction in healthy infants with AB. The subgroup analysis demonstrated that the use of novel imaging techniques resulted in a higher and consistent detection of myocardial dysfunction. MPI (myocardial performance index); TDI (tissue Doppler imaging); (STE (speckle-tracking echocardiography).

Four investigations [26,28,29,30] detected MD with a pooled estimate measure of 5% (CI 95%, 1–9%). There was significant heterogeneity among these studies (Q test; I^2^ = 71.9%, *p* < 0.001). A subgroup analysis was performed based on the echocardiographic methods used to assess MD. After excluding those studies that only used conventional echocardiographic methods to assess MD (LVEF, LVFS, or TAPSE), the pooled incidence of MD was higher (22% CI 95%, 14–29%), and no significant heterogeneity across the studies was observed (I^2^ = 6.1%, *p* = 0.345) (Figure 4). There was no evidence of publication bias when assessed by Egger’s test (*p* = 0.545; Table 5).

#### 3.4.2. Association of Myocardial Strain and Severe AB

**Figure 5:**
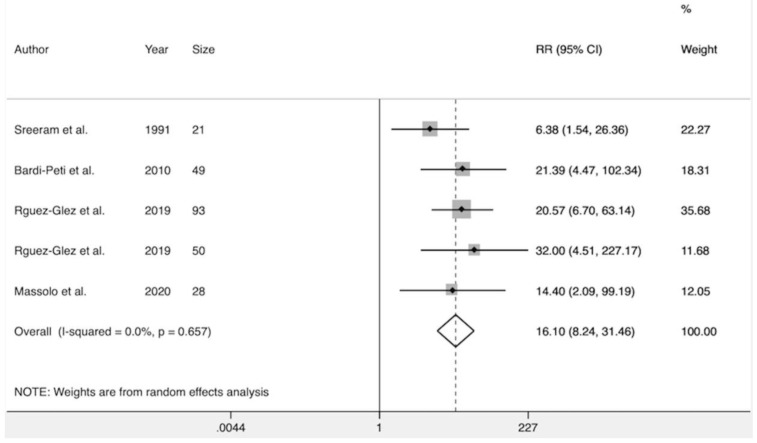
Forest plot showing the association between myocardial strain and the risk of severe AB.

Five studies [22,25,28,29,30] investigated the association between the presence of any type of echocardiographic alteration and the development of severe AB. Pooled results suggested that MS was significantly associated with the risk of severe AB (RR 16.1, CI 95%, 8.2–31.5) (Figure 5). There was no heterogeneity among these studies (Q test; I^2^ = 0%; *p* = 0.657). There was no evidence of publication bias when assessed by Egger’s test (*p* = 0.951; Table 5).

#### 3.4.3. Sensitivity Analysis

The sensitivity analysis results suggested that no individual studies significantly affected the pooled effect of the detection rate of MS, PH, and MD, nor the association between the presence of MS and risk of severe AB, indicating our statistically robust results.

## 4. Discussion

This meta-analysis focused on the incidence and the role of MS as a prognostic factor in healthy infants with AB. To date, there are very few published studies on this subject. Nevertheless, our results suggest that the presence of MS is not infrequent in healthy infants with AB. As most studies performed echocardiography within 24 h of admission, it could be concluded that the MS is present at early stages of the disease in AB. Of note, the exclusion of patients with CHD from this review highlights that MS is not exclusively associated with this condition as traditionally thought. This meta-analysis also provides some evidence that MS could have a role as a predictor for adverse outcomes in this setting. 

### 4.1. Analysis of the Studies that Did Not Detect Myocardial Strain in AB

Three of the studies included did not report any MS in this population [21,24,27]. Some factors could explain this finding. The three studies utilized parameters obtained by conventional echocardiography, which has significant limitations in the assessment of MD and PH. Horter et al. [27] and Esposito et al. [24] did not assess pulmonary pressures in their investigations. Pahl et al. [21] utilized only the measurement of systolic time intervals and not the preferred TRJG. Right ventricular systolic time intervals cannot predict pulmonary artery pressure in cases of mild PH that has been shown in most studies [31]. The complex geometry of the RV limits echocardiographic quantification of function, and often a qualitative assessment is utilized [32]. The TAPSE is the preferred parameter to assess the right ventricular systolic function, but only Horter et al. [27] utilized it and did not find differences between infants that required oxygen therapy and those that not. However, at the time of data collection, all participants presented a mild form of the disease based on the clinical score and no participant required ARS. This fact limits the capacity of this study to establish that there is not MS in any AB, only in mild forms of AB. Pahl et al. [21] and Esposito et al. [24] did not assess the right ventricular function of their participants. Besides, the three studies utilized standard measures of left ventricular function such as M-mode-derived LVFS. Remarkably, this parameter is dependent on the geometry of the ventricles and may be inaccurate in cases of AB with PH due to an anomalous interventricular septal configuration.

### 4.2. Incidence of Myocardial Strain in AB

The presence of acute lung injury secondary to AB can lead to critical cardiovascular effects in this population, especially raising pulmonary vascular resistance, PH, and overloading the RV [33,34]. It is well known that right and left ventricles (LV) operate as an entirety, so the function of one ventricle may influence that of the other, which is well recognized as ventricular interdependence [35,36]. The interventricular septum (IVS) plays a crucial role in mediating ventricular–ventricular interactions. As it shares fibers with both ventricles, the IVS is subject to interventricular pressure gradients and directly impacts on biventricular geometry. Likewise, right ventricular loading, geometry, and function impact left ventricular function and vice versa.

#### 4.2.1. Pulmonary Hypertension

The most common type of echocardiographic alteration found in AB was PH, which occurred consistently in seven studies [22,23,25,26,28,29,30] with a combined incidence of 20%. Only Fitzgerald et al. [23] reported an extremely high incidence of PH in this population (66%). However, the size of the study (*n* = 6) was minimal, all the participants presented a severe clinical course and needed ARS, and the echocardiography was performed late when the patients were ventilated. Therefore, there was an evident selection bias that could lead to overestimation of the real rate of PH in AB. When detected, PH was mild and lower than the artificial pressure in all studies. The maximum reported systolic pulmonary pressures estimated by TRJG ranged between 46–58 mmHg, which means mild-moderate PH. A significant limitation in almost all studies is that the estimation of pulmonary pressures by the TRJG, the standard method to assess pulmonary pressures by echocardiography, was not possible due to the low rate of adequate TRJ. Bardi-Peti et al. [25] found an adequate TRJ in only 2% of the population, Thorburn et al. [26] in 24%, Massolo et al. [30] in 32%, and Sreeram et al. [22] and Rodriguez-Gonzalez et al. [28] in 50% of cases. Fitzgerald et al. [23] found an adequate TRJ in 100% of cases, but we have mentioned the limitations of this study. Thus, some investigators assessed pulmonary pressures by different semi-quantitative and qualitative methods. Bardi-Peti et al. [25] used the ATET ratio, Massolo et al. [30] the LVEI and the SF, and Rodriguez-Gonzalez et al. (REFF) utilized a combination of the above three parameters to define PH. Of note, the subgroup analysis demonstrated a higher detection rate of PH (27%) with a high degree of consistency between these three authors, regarding those studies that estimated pulmonary pressures only by TRJG. However, only the ATET has shown strong correlations with the gold-standard method, the right heart catheterism. Therefore, the classification of patients as PH could be wrong in some cases, making it difficult to establish the real incidence of PH in this population. 

#### 4.2.2. Myocardial Dysfunction 

MD was detected by only four studies with a combined incidence of 5%. In one of these studies [28] MD was observed in only 2% of the cohort. The other three studies [26,29,30] consistently reported a combined incidence of MD of approximately 22%, as shown by the subgroup analysis, and the most remarkable difference between those studies that detected higher incidences of MD and those that did not was the imaging technique used to assess the ventricular function. Seeram et al. [22], Fitzgerald et al. [23], and Bardi-Peti et al. [25] utilized conventional echocardiography and M-mode derived LVSF and did not find MD. Rodriguez-Gonzalez et al. [28] utilized conventional echocardiography and M-mode derived LVSF and TAPSE, and found 2% of RV dysfunction. Thorburn et al. [26] utilized conventional echocardiography and PW-Doppler derived MPI, and found a rate of 20% of RV failure. Rodriguez-Gonzalez et al. [29] in a second study assessed the myocardial function through the MPI derived from TDI, and reported RV and LV dysfunction in 18% and 20% of patients, respectively. Recently, Massolo et al. [30] utilized STE and determined that biventricular dysfunction was present in up to 32% of cases of AB. These results suggest that the MD present in AB is of a mild-grade and subclinical in most cases. Detection of subclinical myocardial changes is difficult to achieve using only conventional echocardiographic techniques that merely evaluate global systolic and diastolic function. Furthermore, given that the interventricular interactions resulting in MD in PH patients are complex, advanced imaging techniques will likely be useful in further elucidating this process. 

TDI was introduced as a new non-invasive simple echocardiographic method for assessment of the ventricular systolic and diastolic functions that can overcome the limitation of the conventional methods. TDI provides quantitative information about myocardial motion with high temporal and spatial resolution and records systolic and diastolic times and velocities within the myocardium and at the corners of the valve annulus [37,38]. As it does not depend on geometrical issues, TDI can be a sensitive and useful tool for the assessment of both diastolic and systolic ventricular function of patients with PH, particularly in light of the inaccuracy of traditional measures of ventricular function. The TDI derived MPI or Tei index is non-invasive, uncomplicated, reproducible, and not significantly influenced by heart rate, ventricular pressure, ventricular dilatation, or importantly, tricuspid regurgitation [37,38]. As the Tei index is essentially a time ratio, it does not depend on ventricular geometry, and therefore it is very useful in the assessment of global right ventricular function in children with a complex right ventricular shape. The index has been shown to correlate well with clinical symptoms of right and left ventricular failure and overall survival, and normal values can be applied to the entire spectrum of the pediatric population with no clinically significant effects of age, heart rate, and body surface area throughout this group of patients [39,40,41,42,43,44,45,46,47,48]. 

Ventricular strain and torsion analysis by STE consist of capturing and tracking points of the two-dimensional echocardiogram throughout the cardiac cycle, generating movement vectors and strain. It has provided additional insight into the changes in systolic function in patients with PH by showing significant reductions in systolic torsion as well as LS and CS [49,50,51,52]. Thus, reliable, quantitative measures of ventricular performance may aid in the detection of MS in AB and to establish a better understanding of disease severity.

It is remarkable that no cases of severe pericardial effusion or left ventricular systolic dysfunction and left ventricular dilation have been found in this systematic review. These findings have been previously reported highlighting the possibility of the occurrence of myocarditis in infants with AB [17,53,54]. However, it has only been reported in case reports and, therefore, these incidental findings would not be a representative picture of the MS in AB.

### 4.3. Myocardial Strain as a Predictor for Adverse Outcomes

In this systematic review, the degree of severity of PH and MD found in healthy infants with AB was mostly mild and subclinical. Nevertheless, myocardial strain seems not to be inconsequential in this setting. PH and MD often complicate any clinical setting when present. Consistently, our results identified a possible association between MS and an increased risk of adverse outcomes in healthy infants with AB (pooled RR 16; CI 95% 8.2–31.5). Sreeram et al. [22] observed that PH was present in all cases with a severe clinical infection. Of note, PH was associated with the only exitus reported by the authors. Fitzgerald et al. [23] reported four infants with PH that presented severe clinical state and required mechanical ventilation and prolonged hospitalization. Bardi-Peti et al. [25] found that PH was associated with the clinical severity of the wheezing episode, and also with a prolonged hospitalization. Thorburn et al. [26] did not communicate differences in the clinical severity or outcomes in their cohort. However, all patients included in the study presented a severe clinical state and were ventilated at the time of the assessment of the MS. Therefore, the MS observed by these authors could be related to the severity of the disease, but it is challenging to state this without a control group with a mild AB. Rodriguez-Gonzalez et al. reported two different cohorts of healthy infants with AB. In the first one [28] they observed that PH was associated with higher clinical scores, hypoxemia, and respiratory acidosis, and that up to 85% of patients with PH required PICU admission and ARS. Of note, these authors observed that the presence of PH was the most important determinant of adverse outcomes in AB. In a second study [29], Rodriguez-Gonzalez et al. found that the presence of LV dysfunction was also associated with clinical severity, hypoxemia, and respiratory acidosis. Of note, 90% of cases with LV dysfunction required PICU admission. Recently, Massolo et al. [30] have reported that patients with biventricular dysfunction presented higher levels of respiratory support. In summary, these findings highlight the relevance of detecting MS in AB, in order to identify high-risk patients for a severe course of the disease.

### 4.4. Cardiac Biomarkers to Assess Myocardial Strain in AB

The results of this systematic review highlight that the detection of MS in AB could be challenging and depends on advanced imaging techniques applied by experts in echocardiography. As these resources are not widely available in the emergency department, it might be useful to identify patients where echocardiographic examination may be warranted. Thus, some authors have investigated the role of cardiac biomarkers in this setting. 

#### 4.4.1. Cardiac Troponin

Cardiac troponin is an inhibitory protein complex forming part of the contractile apparatus of all striated muscle, including the heart. The subtypes troponin T and I are cardio-specific and a highly sensitive marker of myocardial damage. Therefore, they have become established as the gold standard biochemical markers for myocardial necrosis in acute coronary syndrome [55]. In children, they have been shown to be highly predictive of symptomatic myocarditis but not related to the grade of MD or outcomes [56,57,58,59,60]. The third-generation assays for cardiac troponin T and I are now so sensitive and specific that a concept of minimal myocardial damage has arisen [55]. Therefore, elevated levels of these enzymes have also been observed in cases of mild PH and RV strain in primary respiratory diseases. The presence of myocardial damage in AB has been documented in case reports of severely ill and ventilated infants with MD cataloged as myocarditis [17,54,61]. Consistently, this elevation of cardiac troponin has been consistently observed by most of the authors that assessed myocardial injury in the studies included in this systematic review. In the absence of clear ranges of normal values for troponin in children, all authors selected a cut-off point of 10 ng/L to define myocardial injury in their studies. Based on this cut-off point, Thorburn et al. [26] reported a rate of myocardial injury of 41%, Horter et al. [27] 23%, and Rodriguez-Gonzalez et al. [28] 14%. Only Esposito et al. [24] did not find myocardial damage in their cohort, but all included cases were of mild severity. Interestingly, only Rodriguez-Gonzalez et al. [28] could demonstrate a correlation between the elevation of troponin and echocardiographic findings of PH, but this association was not maintained in the multivariate analysis. Therefore, the level of cardiac troponin did not seem to predict MS by echocardiography in AB. 

#### 4.4.2. NT-ProBNP

NT-proBNP is a hormone synthesized and released by ventricular myocytes in response to myocardial overload conditions that increase the myocardial wall strain [62]. NT-proBNP has been established as an adequate biomarker for heart failure, MD, and PH in both adults and children [63,64,65,66,67,68,69,70]. To date, limited work has been done on the application of NT-proBNP in children with AB, and the quality of evidence is low, suggesting that NT-proBNP has the potential to be a useful biomarker in severe bronchiolitis [71,72,73]. In this systematic review, only Rodriguez-Gonzalez et al. [28,29] have attempted to test the diagnostic accuracy of NT-proBNP to predict MS by echocardiography with excellent results. These authors developed two consecutive investigations. In the first [28], they defined elevated NT-ProBNP levels as the 75th percentile of the entire cohort (1635 pg/mL). They found that elevated NT-ProBNP was an adequate predictor for PH assessed by echo, with an AUC of 0.932. They established the best cut-off point at 1345 pg/mL, yielding a sensitivity of 86% and a specificity of 89%. In the second study, they found that NT-ProBNP was also an accurate predictor of LV dysfunction by echo. This time, the best cut-off point selected was 1500 pg/mL that yielded a sensitivity of 80% and a specificity of 95%. Both studies highlighted the high negative predictive value of NT-ProBNP in detecting MS in AB. Therefore, echocardiography should not be necessary for those infants with AB and levels of NT-ProBNP below the cut-off points mentioned. 

### 4.5. Limitations

Our systematic literature retrieval resulted in a limited number of studies and patients and this might result in a lack of statistical power to detect significant differences in the size effects calculated. This could be due to the fact that AB is a primary respiratory condition and few studies have been carried out about the involvement of the heart in those infants without CHD, a high-risk condition for adverse outcomes. Seven of the studies analyzed were classified as fair quality; therefore, our results could be biased by inherent limitations in the original studies. For example, echocardiography is a test with a certain degree of subjectivity, but only one study provided data about the reproducibility of echocardiographic results. Most of the studies were not controlled, and any study could demonstrate the absence of MS previously to the inclusion of the participants. Therefore, the classification of patients as having MS or not could be wrong, in some cases, impacting our results. Although publication bias was assessed, it is important to note that the tests used have low power and at least 10–20 studies are needed to draw conclusions. So publication bias could not be excluded in this systematic review. Combining studies that used different echocardiographic parameters to assess MS could have led to a certain degree of heterogeneity, which was addressed by combining only studies with similar methodologies. The included studies lacked long-term follow-up and outpatient cases. Therefore, their inferences can only be applied to short-term outcomes in hospitalized patients. Meta-analysis on the role of cardiac biomarkers was not possible because no more than one study used them in the same way. Although NT-proBNP could be an accurate predictor for MS in AB, these results should be interpreted cautiously. Finally, the research outcome in assessing predictive value of the myocardial strain as a prognostic factor in AB also differed among the studies. This fact limited the comparison between studies and the generalization of the results. 

## 5. Conclusions and Future Perspectives

The presence of MS in AB is not exclusive to infants with CHD. The results of this systematic review and meta-analysis show that PH and secondary MD are also present in previously healthy infants. Remarkably, the MS is just present in the early stages of the disease and seems to be relevant, as it is related to a worse clinical state and adverse outcomes. Therefore, MS could be a promising early risk factor of severe AB, even in healthy infants. At this moment, there is not enough evidence to rely on cardiac troponin as a diagnostic marker of patients with MS, but there is evidence to support that NT-proBNP could be a promising biomarker for MS in this population. Because the detection of MS in this setting requires usually advanced and not widely available imaging techniques, such as TDI and STE, the measurement of this biomarker is likely to help screen those patients in which an echocardiogram should be warranted. Nevertheless, available data are limited by relatively small sizes, the different imaging methods and cardiac biomarkers employed to assess MS, and the heterogeneity of the research outcomes of the currently available studies. Therefore, sufficiently powered prospective cohort studies with a similar methodology (preferably employing TDI or STE and cardiac biomarkers) and the same research outcomes are needed to conclusively address the usefulness of the assessment of MS in AB. Meanwhile, the findings of this systematic review could throw new insights on the pathogenesis of cardiovascular involvement during this primary respiratory condition and further define the best approach to the disease.

## Figures and Tables

**Table 1:** Downs and Black Quality Assessment Checklist evaluations for methodological quality.

Article	Reporting	External Validity	Internal Validity	Confounding & Selection Bias	Power	Total
Pahl et al. [21]	8/10	2/3	5/7	3/6	0/1	18
Sreeram et al. [22]	8/10	1/3	5/7	3/6	0/1	17
Fitzgerald et al. [23]	8/10	1/3	5/7	2/6	0/1	16
Esposito et al. [24]	9/10	1/3	5/7	3/6	0/1	18
Bardi-Peti et al. [25]	8/10	1/3	5/7	3/6	0/1	17
Thorburn et al. [26]	9/10	1/3	5/7	2/6	0/1	17
Horter et al. [27]	9/10	1/3	5/7	3/6	0/1	18
Rguez-Glez et al. [28]	9/10	2/3	6/7	4/6	0/1	21
Rguez-Glez et al. [29]	9/10	2/3	6/7	4/6	0/1	21
Massolo et al. [30]	9/10	2/3	6/7	3/6	0/1	20

Methodological quality was excellent if total score resulted 26–28; good if total score resulted 20–25; fair if total score resulted 15–19; and poor if total score resulted <14.

**Table 2:** Baseline characteristics of the studies included in this systematic review.

Author (Year)	Study Design & Duration	Number & Age of Participants	Characteristics of Participants & Setting	Rate of Severe AB	Echo Technique & Timing	Biomarker	Rate of MS
Pahl et al. [21]	Prospective not controlled. (?)	19 AB. Age 4.4 m (3 w–10 m)	17 healthy and 2 cystic fibrosis CHD excluded. Setting not mentioned	10/19 (52%)	Conventional 2D doppler echo Within 72 h of admission	No	No
Sreeram et al. [22]	Prospective not controlled. (?)	21 AB. Age (1–24 months)	20 healthy infants & 1 Down Sd. CHD excluded. Setting not mentioned	4/21 (16%)	Conventional 2D doppler echo Within 24 h of admission	No	Yes (24%)
Fitzgerald et al. [23]	Prospective not controlled. (3 m)	6 AB. Age 13 (4–24) w	4 premature & 2 term infants admitted at NICU CHD excluded	6/6 (100%)	Conventional 2D doppler echo Within 72 h of intubation	No	Yes (66%)
Esposito et al. [24]	Prospective not controlled (2 y)	69 AB. Age 4.2 (2.5) m	All healthy infants admitted at pediatric ward CHD excluded	0/69 (0%)	Conventional 2D doppler echo Within 24 h of admission	Yes	No
Bardi-Peti et al. [25]	Prospective controlled (2 y)	49 AB & 62 age-matched controls Age 1–12 m	All healthy infants admitted at pediatric ward CHD excluded	17/49 (34%)	Conventional 2D doppler echo Within 24 h of admission	No	Yes (36%)
Thorburn et al. [26]	Prospective not controlled (2 y)	34 AB. Age 1.4 (0.4–11.7) m.	All healthy infants admitted at PICU	34/34 (100%)	Conventional 2D doppler echo Within 24-48 h of admission	Yes	Yes (24%)
Horter et al. [27]	Prospective not controlled (2 y)	26 AB. Age 2 (1–24) m.	26 healthy infants admitted at pediatric ward CHD excluded	0 (26%)	Conventional 2D doppler echo Within 24 h of admission	Yes	No
Rguez-Glez et al. [28]	Prospective not controlled (3 y)	93 AB. Age 2 (1–10) m.	All healthy infants admitted at pediatric ward CHD excluded	21/93 (22%)	Conventional 2D doppler echo Within 24 h of admission	Yes	Yes (22%)
Rguez-Glez et al. [29]	Prospective controlled (1 y)	50 AB & 50 age-matched controls Age 2 (1–6.5) m.	All healthy infants admitted at pediatric ward CHD excluded	10/50 ((20%)	Conventional & TDI-echo Within 24 h of admission	Yes	Yes (28%)
Massolo et al. [30]	Prospective controlled (5 m)	28 AB & 10 age-matched controls Age 30 (20) d.	All healthy infants admitted at pediatric ward CHD excluded	10/28 (35%)	Conventional & STE-echo Within 24 h of admission	No	Yes (32%)

Abbreviations: ? (Information not available); m (months); y (years); w (weeks); CHD (congenital heart diseases); NICU (neonatal intensive care unit); PICU (pediatric intensive care unit); AB (acute bronchiolitis); TDI (tissue Doppler imaging); STE (speckle-tracking echocardiography). MS (myocardial strain).

**Table 3:** Echocardiographic characteristics of studies that found myocardial strain in AB and the relationship with the clinical state and adverse outcomes.

Author (Year)	Echo Parameters	Incidence & Type of Myocardial Strain	Significant Outcome
Sreeram et al. [22]	LVFS, TRJG, PVV	Mild PH in 23%. No CD	PH associated with 100% of severe infections and 100% exitus (1 patient with Down syndrome and PH). PH disappeared with clinical improvement.
Fitzgerald et al. [23]	LVFS, TRJG	Mild PH in 66%. No CD	100% of patients with PH presented a severe disease and adverse outcomes.
Bardi-Peti et al. [25]	TRGJ, ATET	PH in 28%. No CD	PH was associated to moderate/severe wheezing episodes and prolonged LOS hospitalization.
Thorburn et al. [26]	LVFS, PW-doppler derived RVMPI, TRJG	RVD in 20% (RVD). No PH	All patients with RVD presented a severe clinical state. There was no difference with those patients without RVD.
Rguez-Glez et al. [28]	LVFS, TAPSE, TRJG, ATET, LVEI, SF	PH 22%. RVD 2%	PH was associated with a worse clinical state and impaired gas exchange. Up to 85% of cases with PH presented significantly adverse outcomes (PICU admission).
Rguez-Glez et al. [29]	LVFS, TAPSE, TRJG, ATET, LVEI, SF, TDI derived RVMPI & LVMPI	LVD in 18%. RVD in 20%. PH 28%	LVD, RVD and PH were associated with a severe clinical presentation and impaired gas exchange. Up to 89% of cases with LVD presented significantly adverse outcomes (PICU admission).
Massolo et al. [30]	LVEF, RVFAC, RVFS, LS, CS, RS, TRJG, LVEI, SF	LVD 32%. BD in 32%. PH in 29%	BD was associated with higher frequency of hypoxia and hypercarbia. BD was associated with higher levels of respiratory support.

Abbreviations: LVFS (left ventricular fractional shortening); LVEF: (left ventricle ejection fraction) TRJG (tricuspid regurgitation jet gradient); PVV (pulmonary valve velocity); ATET (pulmonary acceleration time/right ventricular outflow ejection time ratio); PW (pulsed wave); RVMPI (right ventricular myocardial performance index or TEI index); LVMPI (left ventricular myocardial performance index or TEI index); TAPSE (tricuspid annular plane systolic excursion); LVEI (left ventricular eccentricity index); SF (septal flattening); TDI (tissue Doppler imaging); RVFAC (right ventricular fractional area change); LS (longitudinal strain); CS (circumferential strain); RS (radial strain); PH (pulmonary hypertension); RVD (right ventricular dysfunction); LVD (left ventricular dysfunction); BD (biventricular dysfunction); PICU (pediatric intensive care unit); LOS (length of stay).

**Table 4:** Data of the studies that utilized cardiac biomarkers to assess the myocardial strain detected by echocardiography.

Author (Year)	Cardiac Biomarker	Values	Correlation with Echo	Details
Esposito et al. [24]	Troponin I (Abbott AxSYM system)	Normal values (0.011 (0.02) IU/L)	No	All cases were mild without need of PICU admission
Thorburn et al. [26]	Troponin T (ELISA, Roche Diagnostics)	cTnT elevated (> 10ng/L) in 41%	No	All cases were severe and required mechanical ventilation and PICU admission
Horter et al. [27]	Troponin T (ELISA, Roche Diagnostics)	cTnT elevated (>10ng/L) in 23%	No	All cases were mild without need of PICU admission
Rguez-Glez et al. [28]	NT-proBNP (ELISA, Roche Diagnostics)	Elevated (>1635 pg/mL) in 25%	Associated with PH	Increased NT-proBNP was an independent and accurate predictor for PH in AB (AUC = 0.932; *p* < 0.001). The optimal cut-off value yielded a sensitivity of 0.86, specificity of 0.89; PPV of 0.70 and NPV of 0.95)
	Troponin T (Abbott AxSYM system)	Elevated (>10ng/L) in 14%	Associated with PH	cTnT was not an independent predictor for PH in the multivariate analysis (*p* = 0.354)
Rguez-Glez et al. [29]	NT-proBNP (ELISA, Roche Diagnostics)	Elevated in patients with CD (2221 pg/mL vs. 377 pg/mL; *p* < 0.001)	Associated with LVD	The diagnostic performance of NT-proBNP to predict LVD in infants with AB resulted in high (AUC = 0.91). The best estimated cut-off value to predict was 1500 pg/mL, with a sensitivity of 0.80, specificity of 0.95, a PPV of 0.80), and an NPV of 0.95.

Abbreviations: cTnT (cardiac troponin T); CD (cardiac dysfunction); PH (pulmonary hypertension); LVD (left ventricular dysfunction); AB (acute bronchiolitis); PPV (positive predictive value); NPV (negative predictive value); PICU (pediatric intensive care unit); AUC (area under the ROC curve).

**Table 5:** Results of publication Bias (Egger´s Test) for the different meta-analysis performed.

Group	*N*	*t-Value*	*P-Value*	CI95%
Combined MS	10	−0.26	0.809	−10.8 to 9.2
Pulmonary Hypertension	10	0.10	0.921	−6.5 to 7
Myocardial dysfunction	10	−0.87	0.545	−132 to 115
Severe AB	5	0.07	0.951	−1.2 to 1.2

MS (myocardial strain); AB (acute bronchiolitis).
